# Separation and Analysis of Boron Isotope in High Plant by Thermal Ionization Mass Spectrometry

**DOI:** 10.1155/2015/364242

**Published:** 2015-12-24

**Authors:** Qingcai Xu, Yuliang Dong, Huayu Zhu, Aide Sun

**Affiliations:** ^1^Shandong Provincial Key Laboratory of Water and Soil Conservation and Environmental Protection, College of Chemistry and Chemical Engineering, Linyi University, Linyi 276005, China; ^2^State Key Laboratory of Isotope Geochemistry, Guangzhou Institute of Geochemistry, Chinese Academy of Sciences, Guangzhou 510640, China

## Abstract

Knowledge of boron and its isotope in plants is useful to better understand the transposition and translocation of boron within plant, the geochemical behavior in the interface between soil and plant, and the biogeochemical cycle of boron. It is critical to develop a useful method to separate boron from the plant for the geochemical application of boron and its isotope. A method was developed for the extraction of boron in plant sample, whose isotope was determined by thermal ionization mass spectrometry. The results indicated that this method of dry ashing coupled with two-step ion-exchange chromatography is powerful for the separation of boron in plant sample with large amounts of organic matters completely. The ratios of boron isotope composition in those plant tissue samples ranged from −19.45‰ to +28.13‰ (total range: 47.58‰) with a mean value of 2.61 ± 11.76‰ SD. The stem and root isotopic compositions were lower than those in flower and leaf. The molecular mechanism of boron isotope may be responsible for the observed variation of boron isotopic composition and are considered as a useful tool for the better understanding of boron cycling process in the environment and for the signature of living systems.

## 1. Introduction

Boron (B) is a critical micronutrient in the growth of plant, which was undoubtedly considered as a part of the structure in the cell wall [1–3]. More increasing evidence was present for a possible role of B in metabolism processes, such as the maintenance of plasma membrane function and several metabolic pathways. Park and Schlesinger [4] reported that most B is fixed into cell wall and is not recycled internally once used by plant, but some B would be emitted to atmosphere in plant aerosol or during the biomass burning. When plant died, the majority is returned to soil. The global uptake of B by plant from soils can be calculated as 4.5 Tg B/yr [4]. All of these indicated that the cycling of B and the equilibrium of B isotope in the process between plant and soil would be changed.

In nature, B has two stable isotopes: ^10^B and ^11^B. Because B shows a large variation in the stable isotopic composition (~90‰) [5–8], B and its isotope have been used to investigate a wide range of geochemical, cosmochemical, and geophysical problems. Recent concerns about the use of B isotope in biological systems have been taken into consideration for its important role in embryonic development and organogenesis in plant growth [1, 3] and its isotopic fractionation in the cycling of B in the uptake by plant from soil and processing within plant by a series of chemical or biochemical reactions.

The presence of lots of organic matters in plant can influence the emission of Cs_2_BO_2_^+^ ion current in the chamber of TIMS [9, 10]. In plant, the appropriate pretreatment of sample should effectively remove organic contaminants and meanwhile keep B isotopic composition stable. The method using Amberliter IRA 743 resin coupled with cation and anion ion-exchange mixing resin developed by Xiao et al. [11] and Wang et al. [12] was applied as a proxy for the separation of B, especially in the water samples. The techniques of microsublimation [13–15] and digestion with H_2_O_2_ were fit for the samples with few organic matters. The wet chemical digestion with HNO_3_/H_2_O_2_ in common was used to digest plant sample; however, this method would produce the isobaric Cs_2_CNO^+^ ion of *m*/*z* 308 and 309, which affected the determination of B isotope. Wei et al. [16] developed a method using HF, H_2_O_2_, and mannitol mixed solution to separate B from the silicate rock sample successfully, and B isotope ratio was determined by multicollector inductively coupled plasma mass spectrometry (MC-ICP-MS). These techniques mentioned above were not suitable to eliminate large number of organic matters in plant sample. Wieser et al. [17] and Serra et al. [18] attempted B isotopic composition for biogeochemical plant-soil interaction and provenance on the coffee bean. However, methods for determining B isotopic composition in different plant tissues are scarce [19].

In this study, a series of dry ashing experiments coupled with ion-exchange resin chromatography were performed to separate B in plant tissue sample. The B isotopic composition in plant tissue was determined by positive TIMS based on Cs_2_BO_2_^+^ ion. The recoveries of B separation in dry ashing, ion-exchange chromatography, and the whole procedure were examined. And the characteristics and fractionation in the B isotope composition of plant tissue samples were investigated and discussed.

## 2. Material and Methods

### 2.1. Plant Sample and Site

To examine the fractionation of B isotopes in different plant species and within plant tissues, plant samples investigated in this study include various tissues of* Swertia mussotii* Franch. and* Halenia elliptica* D. Don collected in the Qinghai-Tibet Plateau area,* Weigela florida* cv. Red Prince and* Echinacea angustifolia* in Shandong area and* Cynomorium songaricum* Ruper. in Inner Mongolia area, China. The samples of* S. mussotii* and* H. elliptica* were collected in September and October (the flowering and fruiting period), 2012, in Yushu and Banma counties of Qinghai, China, respectively. The root holoparasite* C. songaricum*, known in Chinese herbal medicine as “suoyang,” is a classic Mongolian pharmaceutical plant and usually parasitizes the roots of* Nitraria* spp. [20].* E. angustifolia* and* W. florida* were collected in June, 2012. The collected samples include the root, stem, leaf, and flower tissues of* W. florida*,* S. mussotii*, and* H. elliptica*, stem, leaf, and flower tissues of* E. angustifolia*, and stem and flower of* C. songaricum.* The information about sample site, plant species, and soil conditions in the regions is summarized in Table 1.

### 2.2. Instruments and Reagents

Hydrochloric acid (Guaranteed Reagent) was redistilled in a sealed vessel to remove the exogenous B. The cesium carbonate (spectroscopic pure) was of 99.994% purity. High-purity graphite was added to a mixture of ethanol solution (80%) to obtain the final solution corresponding to 13 mg/g graphite. The isotopic reference standard used in this study was NIST SRM 951 boric acid (Gaithersburg, MD, USA). A solution of mannitol of 1.82% (w/v) and Cs_2_CO_3_ solution containing 12.3 mg/mL of Cs^+^ was also prepared. Sodium carbonate, ammonia hydroxide, and sodium chloride were of the analytical grade reagent. Borax and boric acid were of Guaranteed grade Reagent.

The resins, B specific resin Amberlite IRA 743, strong cation exchange resin Dowex 50W X8, and weakly anion exchange resin Amberlite IRA 67, were purchased from Sigma-Aldrich Co. LLC, China.

High purity water with a B blank less than 0.008 *μ*g was redistilled by subboiling distillation and passed through a resin column filled with B specific resin (Amberlite IRA 743), which was used to prepare the standard solution and working solution.

An inductively coupled plasma optical emission spectrometer (ICP-OES, Vista MPX, Varian, USA) with a 40 MHz radio frequency generator and a charge coupled device detector (Vista Chip) was used to detect B.

### 2.3. Separation of B

Dry ashing was also used to decompose plant sample to eliminate the organic impurities [19]. Traditionally, plant sample was decomposed using wet chemical digestion method of HNO_3_/H_2_O_2_, which can lead to the formation of the isobaric interference Cs_2_CNO^+^ ions of 308 (^133^Cs_2_^10^BO_2_^+^) and 309 (^133^Cs_2_^11^BO_2_^+^) in the ionization chamber in TIMS. About 0.3–0.5 g of dried plant sample was weighted and placed into a quartz crucible. The crucible together with plant sample was placed in a closed microwave-assist Muffle burner. To avoid the bubbling in sample from rapid heating, at first the temperature was raised to 200°C for 1 h for the carbonization of organic matter. Then, the temperature was raised to 550°C for 4 h until the ash was whitish to black. After cooling down, 1 mL of 0.5 mol/L HCl solution was used to dissolve the ash and the solution was transferred to a polypropylene tube.

The B specific resin was used to selectively extract B and to remove the remaining impurities meanwhile. The pH in the sample solution was adjusted to 8-9 using 0.1 mol/L NH_3_·H_2_O solution and then transferred to the conditioned B specific resin at a flow rate of 2.5 mL/min. After rinsing with ultrapure water, 10 mL of 0.1 mol/L HCl at 75°C was used to elute B in the resin; then, the collected eluates were evaporated under a clear air flow at 60°C until 0.5 mL solution was left.

Finally, the 0.5 mL concentrated solution was transferred to a mixing ion exchange resin column, which consists of a strong cation exchange resin (200–400 mesh) and a weak anion exchange resin (100–120 mesh). Fifteen mL of ultrapure water was used to rinse the mixing resin. All collected eluates were transferred to a 15 mL Teflon beaker. Following the addition of Cs_2_CO_3_ solution and mannitol solution, evaporation of the solution continued until about 0.2 mL solution was left. The solution was transferred to a 0.5 mL centrifuge tube and continued evaporating to the approximate concentration of 1 mg B/mL. The solution was stored at 4°C for mass spectrometric analysis.

An outline of the general workflow of B separation in plant sample mentioned above is expressed in Table 2.

### 2.4. Isotopic Measurement of B

The measurement of the B isotopic ratio was performed using a triton TIMS (Thermo Fisher Scientific Inc., USA), which was equipped with a special double cup system allowing static multicollection of Cs_2_^11^BO_2_^+^/Cs_2_^10^BO_2_^+^ (309/308) ions [21, 22]. Tantalum filament was degassed at a current of 3 A for 1 h and then coated with a 2.5 *μ*L of graphite slurry solution. When the slurry solution was almost dried, 1 *μ*L sample solution was loaded on top of the graphite and evaporated to dryness. The determination of B isotope followed the method reported by Xiao et al. [22]. The ^11^B/^10^B ratio was calculated as *R*_309/308_ − 0.00078 [23, 24].

The B isotopic composition of the sample is expressed as *δ* ^11^B (‰) relative to that of the NIST SRM 951 standard:

(1) δ11B‰=B11/B10SamB11/B10Std−1×1000.

Here, (^11^B/^10^B)_Sam_ and (^11^B/^10^B)_Std_ were the B isotopic ratio of sample and NIST SRM 951 H_3_BO_3_, respectively. The measured average ^11^B/^10^B ratio of NIST SRM 951 H_3_BO_3_ was 4.0564 ± 0.0098 (2*σ* = 0.03%, *n* = 5).

## 3. Results and Discussion

### 3.1. Recovery of B in the Dry Ashing

Most attention has been given to the B loss and subsequent isotope fractionation in dry ashing and two-step ion-exchange chromatography in biological samples [19]. The B concentration in plant sample was determined using ICP-OES. Wang et al. [12] and Xiao et al. [11] have reported the effects of pH, acidity and salinity, and temperature of HCl solution on the recoveries of B using Amberlite IRA 743 resin combine with mixing ion-exchange resin. The results indicated that the recovery of B in the two-step ion-exchange chromatography reached to 100%, which means there is no B loss in the process. So, in the following experiments, the recoveries of B in dry ashing and in whole procedure were investigated. To examine the B recovery in dry ashing, borax was used as the reference standard to evaluate the loss of B and the isotopic fractionation in the process. In the five replicates, the mean recovery of 100.2% was obtained, which means that there was no loss of B except the crystal water lost. However, when H_3_BO_3_ was used for the recovery test in dry ashing, there was no B remaining in the tube, which indicated that all B in the format of H_3_BO_3_ would evaporate to air. This suggests that when drying ash at temperatures less than 550°C, B in the form of borate will not lose in the sample, which indicated the conservative behavior of B. Concurrently, low oxygen supply appears to inhibit the variation of B formation in dry ashing.

### 3.2. Recovery of B in Whole Procedure

**Figure 1:**
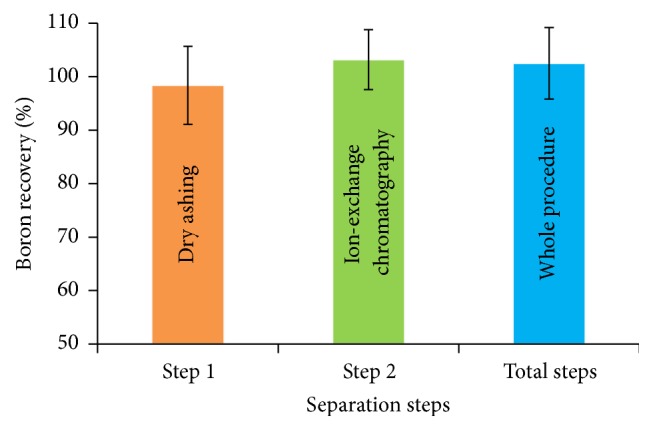
Recovery yields of B for the individual step and the entire procedure.

The two-step ion exchange chromatography method used for B extraction from environmental sample is based on the modification reported by Wang et al. [12] and Xiao et al. [11]. The introduction of the B blank in dry ashing and the chromatography may change the recovery and isotopic composition of B. Reference standard borax was used to pass through the procedure to quantify B recovery and to explore the fractionation of B isotope. The total B blank demonstrated here was 0.008 *μ*g B, which indicates that the variation of B isotopic composition in the procedure should be within the external precision of the isotopic measurement and therefore ignored. The results showed that the recovery of B in the whole procedure ranged from 95.6 to 110.2%, suggesting B was not lost in the process. The comparison of the B isotopic composition in borax before (*δ* ^11^B = 5.08‰, *n* = 5) and after the procedure (*δ* ^11^B = 5.10‰, *n* = 5) with an error deviation of 0.35‰ suggests B isotopic composition was not fractionated in the procedure. The recovery yields of B in the complete procedure as well as for two-step ion-exchange resin chromatographic separation of born in the matrix are shown in Figure 1.

### 3.3. Amount Variation of B in Tissue

**Figure 2:**
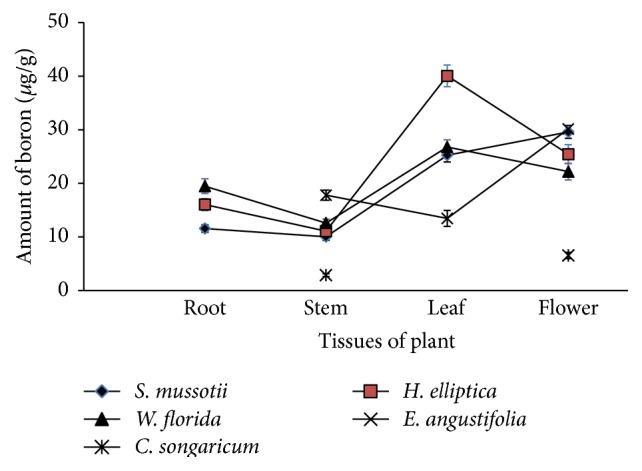
Amounts of B in different plant tissue.

The dried amount of B in plant tissue samples ranged from 2.87 to 40.6 *μ*g/g. The results listed in Figure 2 showed that the dried amount of B was lowest in the stem and highest in flower. In general, the mean dried amounts of B in leaf (26.39 *μ*g/g) and flower (26.85 *μ*g/g) were larger than those in root (15.71 *μ*g/g) and stem (12.89 *μ*g/g). In calculating the average of B in stem and flower, the results of* C. songaricum* were not taken into consideration for the different behavior of B in dried amounts of stem and flower. The dried amounts of B in these plant tissue samples were similar to those in other plants (10–100 *μ*g/g) based on the dry weight reported by Power and Woods [25]. In vascular plants, B moves from the roots with transpiration, accumulating in the growing points of leaves and stems [1, 26]. In most plant species, the B requirement for reproductive growth is higher than that for vegetative growth [27]. Huang et al. [28] demonstrated that B was transported from leaves into actively growing reproductive organs in white lupin (*Lupinus albus*). These findings suggest that, during plant growth, especially in the flowering stage and fruiting period, a large amount of B is needed to promote growth and development of plant reproductive tissues, which leads to the accumulation of B in leaves and flowers.

### 3.4. Fractionation Variation of B Isotopic Composition

**Figure 3:**
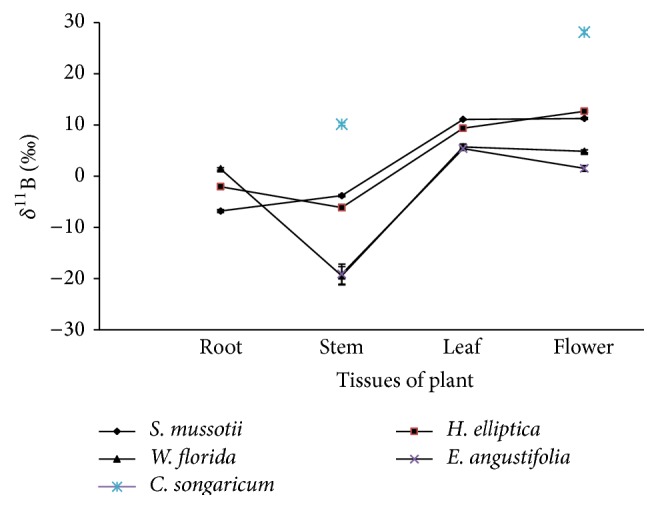
Variation of *δ* ^11^B in the plant tissues.

The *δ* ^11^B values in the tissue of these plant samples are shown in Figure 3. The *δ* ^11^B (‰) values range from −19.45‰ to +28.13‰ (total range: 47.58‰), with a mean value of 2.61 ± 11.76‰ SD. When* C. songaricum* was taken into consideration for calculation, the *δ* ^11^B values range from −19.45‰ to 12.09‰ (range scope: 31.54‰), with a mean of 0.4 ± 10.10‰ SD. These results were similar to those reported by Serra et al. [18] with the *δ* ^11^B values from −11.60‰ to 17.20‰ (mean value: 4.23 ± 7.57‰ SD). Except for the stem and flower tissue of* C. songaricum* (Figure 3), the maximum mean *δ* ^11^B values occurred in the leaf (+7.58‰) and flower tissue (+7.90‰), with the minimum *δ* ^11^B occurring in stem (−12.11‰) and then in root (−2.74‰).

In Figures 2 and 3, the variation of *δ* ^11^B is similar to that of dried amount of B, which may be relative to the uptake and transportation of B within plant. In plant growth, the uptake of B by root is in the form of B(OH)_3_ or B(OH)_4_^−^ [29]. B(OH)_3_ can be taken up by plants through passive diffusion or an energy dependent high-affinity transport system [30]. Borate can form the most stable diesters with cis-diols on a furanoid ring [1, 27]. The movement of B depends on the sugar or polyol transport molecules [1].

The heavier isotope of B is favored in B(OH)_3_ and B(OH)_4_^−^ is depleted in ^11^B; thus, the variation of *δ* ^11^B in root is lower and closely associated with that in the soil [31]. B can be also transported to reproductive and vegetative tissues [30, 32], although this capacity varies among species [33, 34]. After B is transported from the root to the stem, the *δ* ^11^B value in the root is greater than that in stem as different velocities influence B mobility by transportation or ion exchange. During plant growth, a large number of sugar alcohols such as sorbitol, mannitol, and fructose, which are utilized by the combination of hydroxyls of B(OH)_3_, are required in the leaves and flowers [35, 36]. Thus, much ^11^B will preferentially go into the leaves and flowers along with B(OH)_3_, resulting in higher *δ* ^11^B values in the leaf and in flower.

### 3.5. Δ*δ* ^11^B Values in Higher Plants

The fractionation between two components *i* and *j* was determined as follows [37]:

(2) $$\begin{array}{c} \Delta \delta^{11}B_{i-j}={\left(\;\delta^{11}B\;\right)}_{i}-{\left(\;\delta^{11}B\;\right)}_{j}. \end{array}$$

**Figure 4:**
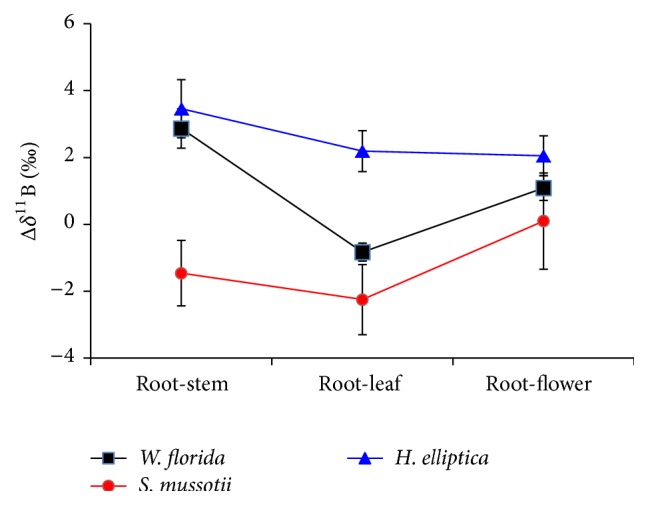
B isotope fractionation in tissues (stem, leaf, and flower) versus root.

The variations of Δ*δ* ^11^B in the plant tissue samples* W. florida*,* S. mussotii*, and* H. elliptica* are illuminated in Figure 4. B isotopic composition is significantly fractionated in the plant species and in different tissues of the same plant. Δ*δ* ^11^B_root-stem_, Δ*δ* ^11^B_root-leaf_, and Δ*δ* ^11^B_root-flower_ in* H. elliptica* were of 3.46‰, 2.19‰, and 2.05‰, in* W. florida* were of 2.87‰, −0.83‰, and 1.09‰, and in* S. mussotii* were of −1.46‰, −2.25‰, and 0.1‰.

The maximum of Δ*δ* ^11^B (>+0‰) was found in the root-stem, root-leaf, and root-flower of* H. elliptica*, which means that relative to that in* W. florida* and* S. mussotii,* more ^11^B was used by* H. elliptica* from the region where it grows. The minimum of those values (<−0‰) observed in* S. mussotii*, which indicates that more ^10^B was accumulated in the tissues of* S. mussotii*, may be caused by the different manner of uptake and transference or by the biophysiological effect of B on plants. This presence would be another important channel for the variation of B isotope composition in the global cycle of B by further uptake and translocation of B in the tissues of plants.

## 4. Conclusions

A proposed method using dry ashing combined with two-step chromatography was developed for the separation of B in plant sample, which enables applications of B isotopes in biogeochemistry, behavior of plant and soil, and plant metabolism. Within the plant, the heavier isotope is enriched in leaf and flower. B isotope is fractionated by the mechanism of diffusion, transportation, or a biological effect between tissues. Moreover, knowledge of cycling and fractionation mechanism of B isotope in the uptake of plants will contribute to better understanding of the global biogeochemical cycle of B.

## Figures and Tables

**Table 1:** Sampling sites and plant species.

Species	Sampling location	Altitude (m)	Longtitude	Latitude	Habit type	Soil type
*W. florida*	Linyi, Shandong	71	118°17′13.21′′E	35°6′21.24′′N	Sand	Shantung soil
*E. angustifofia*	Pingyi, Shandong	242	117°4023.52′′E	35°15′56.88′′N	Sand	Cinnamon soil
*C. songaricum*	Jilantai, Inner Mongolia	1060	105°37′13.83′′E	39°34′42.61′′N	Sand	Sandy soil
*S. mussotii*	Yushu, Qinghai	3585	97°53′23.28′′E	33°20′12.12′′N	Shrub grassland	Alpine steppe soil
*H. elliptica*	Banma, Qinghai	3514	100°47′3.48′′E	32°46′27.12′′N	Bottomland meadow	Meadow soil

**Table 2:** Workflow of the separation of B in plant sample.

Workflow	Procedure	Validation
Decomposition of plant sample	Dry ashing	Recovery test with Ref. Std.

B separation (Two-step ion-exchange resin chromatography)	Amberlite IRA 743 resin	Recovery test with Ref. Std.
	Mixed ion-exchange resin	Isotope fractionation test with isotopic Ref. Std.

Determination of B isotope	Cs_2_BO_2_^+^-graphite technique for TIMS	Isotope fractionation test with isotopic Ref. Std.

Ref. Std.: Reference Standard.

## References

[1] Blevins D. G., Lukaszewski K. M. (1998). Boron in plant structure and function. *Annual Review of Plant Physiology and Plant Molecular Biology*.

[2] O'Neill M. A., Ishii T., Albersheim P., Darvill A. G. (2004). Rhamnogalacturonan II: structure and function of a borate cross-linked cell wall pectic polysaccharide. *Annual Review of Plant Biology*.

[3] Shaaban M. M. (2010). Role of boron in plant nutrition and human health. *American Journal of Plant Physiology*.

[4] Park H., Schlesinger W. H. (2002). Global biogeochemical cycle of boron. *Global Biogeochemical Cycles*.

[5] Aggarwal J. K., Palmer M. R. (1995). Boron isotope analysis. A review. *The Analyst*.

[6] Barth S. (1993). Boron isotope variations in nature: a synthesis. *Geologische Rundschau*.

[7] Bassett R. L. (1990). A critical evaluation of the available measurements for the stable isotopes of boron. *Applied Geochemistry*.

[8] Palmer M. R., Swihart G. H. (1996). Boron isotope geochemistry: an overview. *Reviews in Mineralogy*.

[9] He M. Y., Xiao Y. K., Jin Z. D. (2013). Quantification of boron incorporation into synthetic calcite under controlled pH and temperature conditions using a differential solubility technique. *Chemical Geology*.

[10] He M. Y., Xiao Y. K., Jin Z. D. (2013). Accurate and precise determination of boron isotopic ratios at low concentration by positive thermal ionization mass spectrometry using static multicollection of Cs_2_BO_2_
^+^ ions. *Analytical Chemistry*.

[11] Xiao Y. K., Liao B. Y., Liu W. G., Xiao Y., Swihart G. H. (2003). Ion exchange extraction of boron from aqueous fluids by amber lite IRA 743 resin. *Chinese Journal of Chemistry*.

[12] Wang Q. Z., Xiao Y. K., Wang Y. H., Zhang C. G., Wei H. Z. (2002). Boron separation by the two-step ion-exchange for the isotopic measurement of boron. *Chinese Journal of Chemistry*.

[13] He M. Y., Xiao Y. K., Ma Y. Q., Zhang Y. L., Xiao J. (2011). Effective elimination of organic matter interference in boron isotopic analysis by thermal ionization mass spectrometry of coral/foraminifera: micro-sublimation technology combined with ion exchange. *Rapid Communications in Mass Spectrometry*.

[14] Sun A. D., Xu Q. C., Xu S. J., Shangguan X., Shen H., Sun J. (2013). Determination of boron using headspace liquid phase micro-sublimation coupled with inductively coupled plasma optical emission spectrometry. *Analytical Letters*.

[15] Wang B. S., You C. F., Huang K. F. (2010). Direct separation of boron from Na- and Ca-rich matrices by sublimation for stable isotope measurement by MC-ICP-MS. *Talanta*.

[16] Wei G. J., Wei J. X., Liu Y. (2013). Measurement on high-precision boron isotope of silicate materials by a single column purification method and MC-ICP-MS. *Journal of Analytical Atomic Spectrometry*.

[17] Wieser M. E., Iyer S. S., Krouse H. R., Cantagallo M. I. (2001). Variations in the boron isotope composition of *Coffea arabica* beans. *Applied Geochemistry*.

[18] Serra F., Guillou C. G., Reniero F. (2005). Determination of the geographical origin of green coffee by principal component analysis of carbon, nitrogen and boron stable isotope ratios. *Rapid Communications in Mass Spectrometry*.

[19] Rosner M., Pritzkow W., Vogl J., Voerkelius S. (2011). Development and validation of a method to determine the boron isotopic composition of crop plants. *Analytical Chemistry*.

[20] Yang Y. C. (1991). *Tibetan Medicines*.

[21] Deyhle A. (2001). Improvements of boron isotope analysis by positive thermal ionization mass spectrometry using static multicollection of Cs_2_BO_2_
^+^ ions. *International Journal of Mass Spectrometry*.

[22] Xiao Y. K., Beary E. S., Fassett J. D. (1988). An improved method for the high-precision isotopic measurement of boron by thermal ionization mass spectrometry. *International Journal of Mass Spectrometry and Ion Processes*.

[23] Chetelat B., Gaillardet J., Freydier R., Négrel P. (2005). Boron isotopes in precipitation: experimental constraints and field evidence from French Guiana. *Earth and Planetary Science Letters*.

[24] Nakano T., Nakamura E. (1998). Static multicollection of Cs_2_BO_2_
^+^ ions for precise boron isotope analysis with positive thermal ionization mass spectrometry. *International Journal of Mass Spectrometry*.

[25] Power P. P., Woods W. G. (1997). The chemistry of boron and its speciation in plants. *Plant and Soil*.

[26] Lovatt C. J. (1985). Evolution of xylem resulted in a requirement for boron in the apical meristems of vascular plants. *New Phytologist*.

[27] Loomis W. D., Durst R. W. (1992). Chemistry and biology of boron. *BioFactors*.

[28] Huang L., Bell R. W., Dell B. (2008). Evidence of phloem boron transport in response to interrupted boron supply in white lupin (*Lupinus albus* L. cv. Kiev Mutant) at the reproductive stage. *Journal of Experimental Botany*.

[29] Jiao X. Y., Zhu Y. G., Jarvis B. C., Quick W. P., Christie P. (2005). Effects of boron on leaf expansion and intercellular airspaces in mung bean in solution culture. *Journal of Plant Nutrition*.

[30] Shelp B. J., Marentes E., Kitheka A. M., Vivekanandan P. (1995). Boron mobility in plants. *Physiologia Plantarum*.

[31] Tanaka M., Fujiwara T. (2008). Physiological roles and transport mechanisms of boron: perspectives from plants. *Pflügers Archiv—European Journal of Physiology*.

[32] Matoh T., Ochiai K. (2005). Distribution and partitioning of newly taken-up boron in sunflower. *Plant and Soil*.

[33] Brown P. H., Shelp B. J. (1997). Boron mobility in plants. *Plant and Soil*.

[34] Goldbach H. E. (1997). A critical review on current hypothesis concerning the role of boron in higher plants: suggestion for further research and methodological requirements. *Journal of Trace and Microprobe Techniques*.

[35] Hu H., Brown P. H. (1996). Phloem mobility of boron is species dependent: evidence for phloem mobility in sorbitol-rich species. *Annals of Botany*.

[36] Hu H., Penn S. G., Lebrilla C. B., Brown P. H. (1997). Isolation and characterization of soluble boron complexes in higher plants. *Plant Physiology*.

[37] Moynier F., Pichat S., Pons M. L., Fike D., Balter V., Albarède F. (2009). Isotopic fractionation and transport mechanisms of Zn in plants. *Chemical Geology*.

